# Lower Urinary Tract Dysfunction Among Patients Undergoing Surgery for Deep Infiltrating Endometriosis: A Prospective Cohort Study

**DOI:** 10.3390/jcm13237367

**Published:** 2024-12-03

**Authors:** Anna-Sophie Villiger, Diana Hoehn, Giovanni Ruggeri, Cloé Vaineau, Konstantinos Nirgianakis, Sara Imboden, Annette Kuhn, Michael David Mueller

**Affiliations:** Department of Obstetrics and Gynecology, Bern University Hospital, University of Bern, 3010 Bern, Switzerland; diana.hoehn@insel.ch (D.H.); giovanni.ruggeri@insel.ch (G.R.); cloe.vaineau@insel.ch (C.V.); annette.kuhn@insel.ch (A.K.); michel.mueller@insel.ch (M.D.M.)

**Keywords:** endometriosis, laparoscopy, lower urinary tract symptoms, pelvic pain, prospective studies, urinary bladder, urodynamics

## Abstract

**Background/Objectives**: Postsurgical lower urinary tract dysfunction (LUTD) is a common problem following deep infiltrating endometriosis (DIE) resection. The condition may be caused either by surgically induced damage to the bladder innervation or by pre-existing endometriosis-associated nerve damage. The aim of this study is to evaluate the efficacy of preoperative and postoperative multichannel urodynamic testing (UD) in identifying pre-existing or surgically induced LUTD among patients with DIE. **Methods**: Women with suspected DIE and planned surgical resection of DIE at the Department of Obstetrics and Gynecology at the University Hospital of Bern from September 2015 to October 2022 were invited to participate in this prospective cohort study. UD was performed before and 6 weeks after surgery. The primary outcome was the maximum flow rate (uroflow), an indicator of LUTD. Secondary outcomes were further urodynamic observations of cystometry and pressure flow studies, lower urinary tract symptoms (LUTS) as assessed by the International Prostate Symptom Score (IPSS), and pain as assessed by the visual analog scale (VAS). **Results**: A total of 51 patients requiring surgery for DIE were enrolled in this study. All patients underwent surgical excision of the DIE. The cohort demonstrated a uroflow of 22.1 mL/s prior to surgery, which decreased postoperatively to 21.5 mL/s (*p* = 0.56, 95%CI −1.5–2.71). The mean bladder contractility index (BCI) exhibited a notable decline from 130.4 preoperatively to 116.6 postoperatively (*p* = 0.046, 95%CI 0.23–27.27). Significant improvements were observed in the prevalence of dysmenorrhea, abdominal pain, dyspareunia, and dyschezia following surgical intervention (*p* = <0.001). The IPSS score was within the lower moderate range both pre- and postoperatively (mean 8.37 vs. 8.51, *p* = 0.893, 95%CI −2.35–2.05). Subgroup analysis identified previous endometriosis surgery as a significant preoperative risk factor for elevated post-void residual (43.6 mL, *p* = 0.026, 95%CI 13.89–73.37). The postoperative post-void residual increased among participants with DIE on the rectum to 54.39 mL (*p* = 0.078, 95%CI 24.06–84.71). Participants who underwent hysterectomy exhibited a significantly decreased uroflow (16.4 mL/s, *p* = 0.014, 95%CI 12–20) and BCI (75.1, *p* = 0.036, 95%CI 34.9–115.38). **Conclusions**: Nerve-respecting laparoscopy for DIE may alter bladder function. UD is not advisable before surgery, but the measurement may detect patients with LUTD.

## 1. Introduction

Lower urinary tract dysfunction (LUTD) may occur following any pelvic surgical procedure [1,2,3]. It is currently unclear whether LUTD among patients with deep infiltrating endometriosis (DIE) is a direct consequence of the disease itself or a sequel of surgical intervention [4]. A number of prospective studies have indicated that preoperative multichannel urodynamic testing (UD) could provide valuable information and have suggested that it should be performed routinely, even among asymptomatic patients [4,5,6]. UD determines the function of the lower urinary tract [7,8,9]. Urodynamic studies usually involve uroflowmetry and post-void residual urine volume (PVR) measurement before filling cystometry and pressure flow studies with an in situ catheter [10]. Filling cystometry measures the bladder storage function and describes bladder sensation, detrusor activity, and bladder capacity. Pressure flow studies define the voiding function in terms of detrusor and urethral function, e.g., by measuring the urine flow rate. The maximum flow rate represents a mixture of detrusor function, pelvic floor relaxation, and urethral function and can indicate impaired bladder function, insufficient pelvic floor relaxation, or other intravesical obstruction. However, UD is an invasive test that is time-consuming, labor-intensive and costly; the benefit to the therapeutic decision must be considered when determining indications.

DIE is observed in approximately 20% of patients with endometriosis and is the most aggressive form of the disease [11]. DIE is characterized by the implantation of endometrial glands and stroma outside the endometrial cavity, with infiltration beneath the peritoneum, causing pelvic pain and infertility [12]. In cases where medical treatment proves ineffective in relieving pain or restoring fertility, laparoscopic excision of endometriotic lesions is recommended [13]. Although DIE is a benign condition, its growth often infiltrates adjacent pelvic structures, frequently into pelvic autonomic nerves, namely the inferior hypogastric plexus (IHP), the inferior hypogastric nerve (IHN), and the pelvic splanchnic nerves (PSNs). Consequently, following surgical excision of DIE, LUTD may occur in up to 40% of cases [14,15,16,17], potentially resulting from surgical damage to the IHP, IHN, and PSNs [18,19]. The objective of the nerve-respecting laparoscopy for DIE is to preserve these nerves whenever possible: the IHN is dissected at the level of the promontory medial to the ureter. In the pararectal fossa, the PSNs are visualized posterolateral and the IHP anteromedial to the deep uterine vein. Alternatively, LUTD may be present prior to surgical intervention: DIE impairs bladder storage and voiding capacity, resulting in peripheral damage to the bladder and detrusor innervation in patients without urinary symptoms [6,20]. Accordingly, the objective of this study was to evaluate pre- and postoperative UD among patients with DIE to identify pre-existing or surgically induced LUTD.

## 2. Materials and Methods

This single-center, prospective, observational cohort study was conducted at the Department of Obstetrics and Gynecology of the University Hospital of Bern (Switzerland) between September 2015 and December 2022. The recruitment and exposure period commenced in September 2015 and finished in October 2022. The follow-up period was completed in December 2022, and the data collection was terminated in April 2023. The Department of Obstetrics and Gynecology of the University Hospital of Bern is a European Endometriosis League (EEL) certified endometriosis center and serves as a tertiary referral center for the treatment of endometriosis. Three experienced surgeons performed the laparoscopic nerve-respecting surgery for DIE. Detailed surgical strategy was tailored to the individual endometriotic lesions and anatomical situation. The objective of the surgery was to remove as many of the endometriotic lesions as possible, with the aim of preserving the IHP and lower plexus wherever feasible. Endometriosis classification was determined based on the #ENZIAN classification of the DIE and the rASRM classification at the time of surgery [21]. “The #Enzian classification is based on the well-known Enzian classification for DIE using three compartments (vagina, rectovaginal space (RVS); uterosacral ligaments (USL)/cardinal ligaments/pelvic sidewall and rectum) as well as so-called distant sites such as the urinary bladder, ureters, and other extragenital lesions. It also includes peritoneal, ovarian and other bowel involvement, as well as adhesions involving the tubo-ovarian unit and, optionally, tubal patency” [21].

We invited eligible women with a clinical or imaging diagnosis of DIE who were symptomatic and required surgical treatment to participate in the study. The necessity for endometriosis surgery was established based on the patient’s symptoms, such as pain or infertility. To substantiate a clinical suspicion of DIE, all participants underwent a standardized assessment, including an advanced pelvic ultrasound scan and, in some cases, magnetic resonance imaging. Information about the study and instructions regarding the clinical examination and interventions were given orally and in a detailed written informed consent document. After thorough counseling prior to any study intervention took place, patients provided written consent. The privacy rights of the human participants were respected.

We excluded patients with bladder endometriosis requiring bladder surgery, prior extensive colorectal surgery, pelvic malignancies, radiation therapy of the pelvis, recurrent urinary tract infections, history of psychiatric or neurologic disease, and concomitant urogenital prolapse greater than stage 1 according to the Pelvic Organ Prolapse Quantification system [22].

The primary outcome measure was the maximum flow rate (uroflow), as determined by UD and expressed in milliliters per second [mL/s]. A flow rate below 15 mL/s was considered indicative of impaired function. Secondary outcomes included a range of cystometry and pressure flow measurements, as well as the assessment of subjective lower urinary tract symptoms (LUTS) rated using the International Prostate Symptom Score (IPSS) and the measurement of pain score by visual analog scale (VAS). All patients in the cohort underwent multichannel urodynamic testing (SEDIA^®^, Giviez, Switzerland) prior to surgery and at the six-week follow-up thereafter. Testing was conducted in accordance with the Good Urodynamic Practices Guidelines of the International Continence Society [10,23]. As UD is not currently the standard of care for these patients, they were neither billed nor compensated for this procedure. Conventional urodynamic studies were conducted to assess the following parameters: filling cystometry (bladder capacity [mL], first sensation of bladder filling [mL], detrusor overactivity [yes/no], and maximum urethral closure pressure [cmH_2_O]) and pressure flow (uroflow, [mL/s], bladder contractility index [BCI = PdetQmax + 5 Qmax], and post-void residual [PVR, mL]). BCI was classified into two categories: regular > 100 and weak < 100 [24]. A significant PVR was defined as above 100 mL. All methods, terms, definitions, and units pertaining to urinary symptoms were in accordance with the standards jointly recommended by the International Continence Society and the International Urogynecological Association [25].

Moreover, the occurrence of symptoms was recorded in advance of and six weeks following surgery using the VAS and IPSS questionnaire. The IPSS is validated for women with LUTS and comprises eight questions (incomplete emptying, frequency, intermittency, urgency, weak stream, straining, nocturia, and quality of life) [26,27,28,29]. It objectively measures the subjective burden of a patient’s LUTS through a 10 min self-administered questionnaire. LUTS were classified according to absent/minimal (IPSS: 0–7), moderate (IPSS: 8–19), or severe (IPSS: >20). The VAS questionnaire comprises five questions and assesses the degree of dysmenorrhea, abdominal pain, dyspareunia, dysuria, and dyschezia during the previous 4 weeks on a scale from 1 to 10, with higher values corresponding to increased symptom severity.

Statistical analysis was conducted using Stata 16^®^ (Stata Corporation, College Station, TX, USA). The median, range, mean, and standard deviation were calculated for continuous variables, while the percentages were calculated for the qualitative variables. The parametric and non-normally distributed continuous variables were analyzed using the paired t-test and Wilcoxon signed-rank test, respectively, in order to achieve the study objectives. To ascertain whether preoperative or postoperative LUTD is associated with specific procedures (e.g., bowel resection) or the location of endometriosis, we evaluated the primary and secondary outcomes in relation to the surgical procedures, employing regression-based methods adjusted for potential confounding variables. Cases with missing values were excluded from the analysis. A significant correlation was identified when the *p*-value was less than 0.05. A priori statistical power analysis was performed to examine the potential impact of bladder denervation on postoperative outcomes, as previously investigated in a related study [30]. In light of the possibility that partial denervation may be a contributing factor to postoperative LUTD following endometriosis surgery, this study was used as a point of reference [30]. Based on a clinically relevant effect size (ES) of 0.6, with an alpha value of 0.05 and a power value of 0.90, the estimated sample size required with this ES was approximately *n* = 33.

Ethical approval for this study was obtained from the Ethics Commission of the Canton of Bern, Switzerland (reference number: 131/15, PB_2016-02013). The study was conducted in accordance with the guidelines set forth in the Declaration of Helsinki (DoH) [31], the Essentials of Good Epidemiological Practice issued by Public Health Switzerland (EGEP) [32], and the standards set forth by Swiss law. This study adheres to the reporting standards set forth in the Strengthening the Reporting of Observational Studies in Epidemiology (STROBE) statement [33,34].

## 3. Results

**Figure 1:**
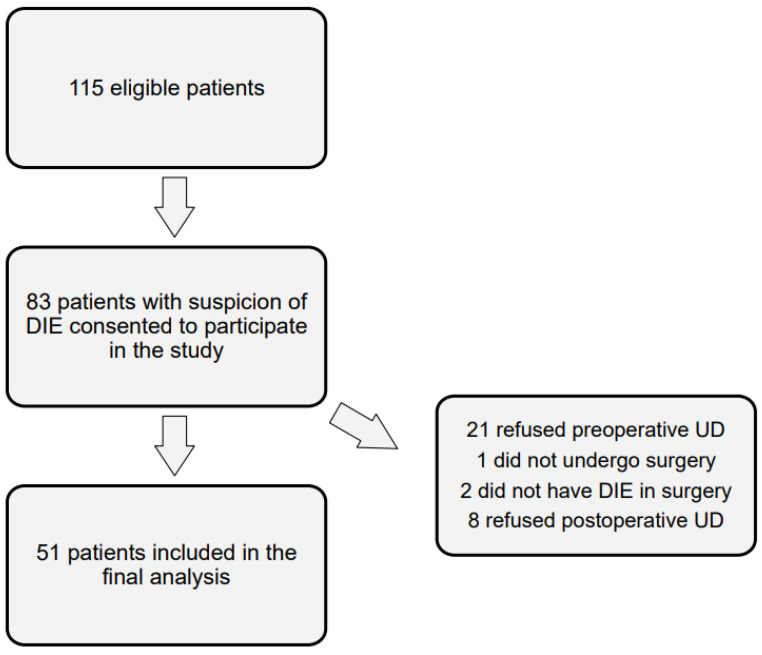
Flowchart of the number of participants at each stage of the study.

In total, 115 patients who met the eligibility criteria were invited to participate in the study during the recruitment phase (Figure 1). A total of 83 patients who were suspected to have DIE consented to participate in this study. Of those, 21 declined the preoperative urodynamic examination, one did not undergo surgery, two did not have DIE in surgery, and eight patients refused to participate in the postoperative UD. Subsequently, 51 patients were included in the final analysis. All participants underwent laparoscopic surgery with excision of DIE in accordance with the principles of nerve-respecting surgery at the endometriosis center of the Bern University Hospital between September 2015 and October 2022.

A summary of the demographic and surgical characteristics of the patients is provided in Table 1. Nineteen women had previously undergone at least one surgical procedure for endometriosis. The USL and the RVS were the most frequently affected anatomic sites, with 44 and 36 cases, respectively. The rectum was the third most affected site, with 25 cases. The majority of patients exhibited involvement of multiple compartments. A minimally invasive surgical technique was employed in all cases. Two patients experienced perioperative complications, including one case of significant vessel injury and one instance of peripheral nerve injury due to compression.

**Figure 2:**
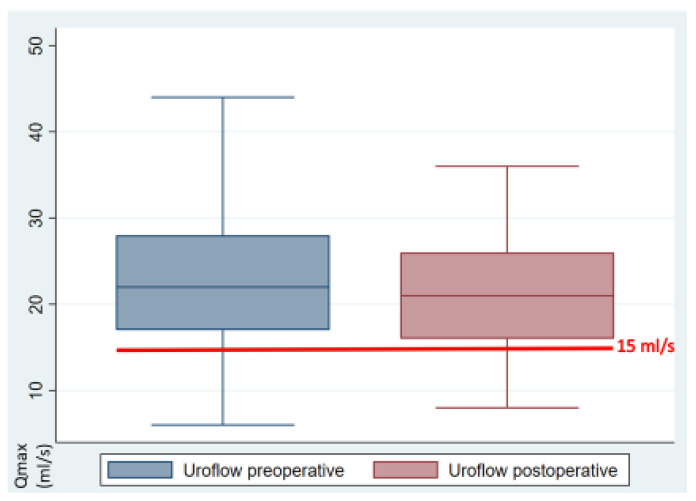
Uroflow of the cohort pre- and postoperatively (mL/s).

**Figure 3:**
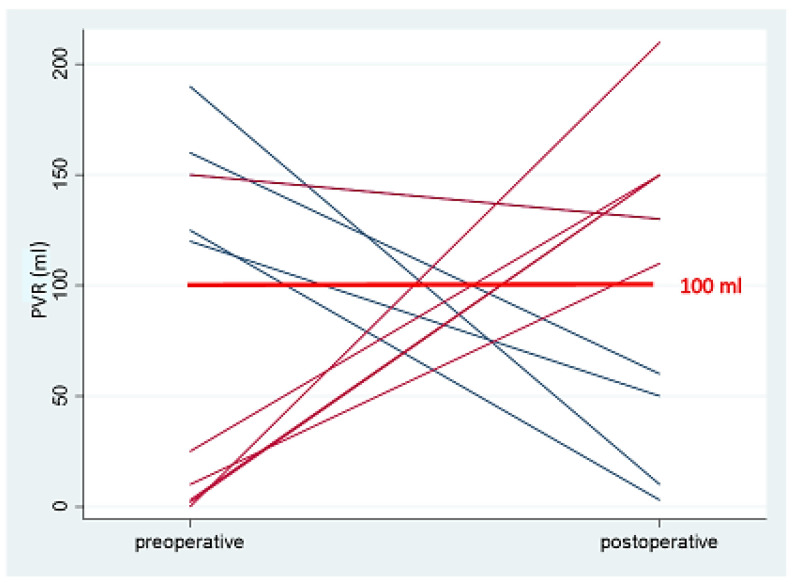
Pathological post-void residual pre- and postoperatively (mL).

A statistical analysis of the preoperative and postoperative outcome parameters is presented in Table 2. The primary outcome, uroflow, was 22.1 mL/s preoperatively and decreased to 21.5 mL/s postoperatively (*p* = 0.56, 95%CI −1.5–2.71). Ten patients exhibited impaired uroflow prior to and following the surgical procedure (Figure 2). The uroflow was observed to be bell-shaped in 40 patients, while 11 patients displayed a multi-peaked, intermittent flow pattern. A statistically significant reduction in BCI was observed, which remained within the normal range, from 130.4 preoperatively to 116.6 postoperatively (*p* = 0.046, 95%CI 0.23–27.27). Nine patients demonstrated a diminished BCI both preoperatively and postoperatively. There was a slight increase in post-void residual (PVR) increased slightly from 26.1 mL to 33.6 mL (*p* = 0.39, 95%CI −24.85–9.87). Prior to the surgical procedure, five patients displayed indications of voiding impairment due to a considerable PVR. Six patients presented with this issue postoperatively, although, with the exception of one patient, these cases were all distinct (Figure 3). The incidence of detrusor contractions during filling exhibited a statistically significant reduction from 15.7% to 4% (*p* = 0.049, 95%CI 0.003–0.23). Maximum urethral closure pressure (MUCP) dropped statistically significantly from 95.4 cmH_2_O to 82.1 cmH_2_O (*p* = 0.003, CI 4.76–21.89). The most severe preoperative symptom was dysmenorrhea, with an average rating of 5.4 on the visual analog scale. All the VAS symptoms exhibited a statistically significant improvement following surgery (*p* < 0.001), with the exception of dysuria, which demonstrated low VAS scores pre- and postoperatively. The IPSS score was within the lower moderate range both pre- and postoperatively (mean 8.37 vs. 8.51, *p* = 0.893, 95% −2.35–2.05). The IPSS scores increased only for the weak urinary stream statistically significantly (*p*= 0.043, 95%CI −1.06–−0.02).

The effect of the DIE extension (#ENZIAN) and the surgery performed on the UD findings was then analyzed (Table S1, Supplementary Materials). No statistical or clinical significance could be observed between DIE extension and performed interventions.

In order to identify the parameters that contribute to the development of pathological preoperative UD findings (Table S2, Supplementary Materials), we observed that patients with adenomyosis exhibited a preoperative reduction in bladder capacity of 254.8 mL (*p* = 0.01, 95%CI 168.41–341.28), which was statistically significant. The results demonstrated that patients with rASRM Stage 1 exhibited statistically significant elevations in uroflow (28.5 mL/s, *p* < 0.001, 95%CI 16.9–40.1), BCI (164, *p* < 0.001, 95%CI 100.07–227.93), and bladder capacity (485 mL, *p* < 0.001, 95%CI 236.38–733.62). Additionally, a statistically significant reduction in MUCP (82.5 cmH_2_O, *p* < 0.001, 95%CI 36.85–128.15) was observed. A statistically significant preoperative risk factor for elevated PVR was identified in patients who had previously undergone surgery for endometriosis (43.6 mL, *p* = 0.026, 95%CI 13.89–73.37).

The postoperative outcomes were correlated with the different surgical procedures, as detailed in Table S3 (Supplementary Materials). A statistically significant increase in residual volume was observed among patients with DIE on the rectum, reaching 54.39 mL (*p* = 0.019, 95%CI 24.06–84.71). Additionally, patients who underwent hysterectomy demonstrated a statistically significant reduction in uroflow (16.4 mL/s, *p* = 0.014, 95%CI 12–20) and BCI (75.1, *p* = 0.036, 95%CI 34.9–115.38).

A further subgroup analysis was conducted with the objective of establishing a correlation between the preoperative and postoperative symptoms and the corresponding urodynamic parameters. No correlative alterations in the urodynamic parameters were observed in relation to specific symptoms, including dysmenorrhea, dysuria, urgency, and incomplete emptying. None of the three patients with preoperative severe IPSS scores indicative of severe pathology demonstrated any pathological findings in the UD. Of the five patients with preoperative pathological PVR, three exhibited impaired uroflow. Two patients with severe IPSS scores revealed impaired uroflow postoperatively; however, neither patient demonstrated pathologic PVR. Among the six patients who presented pathological PVR postoperatively, two demonstrated impaired postoperative uroflow. A single patient with a pathological postoperative PVR displayed both an impaired uroflow and PVR in the preoperative measurements.

## 4. Discussion

The current study proves that 20% of patients with DIE present with LUTD prior to surgical intervention. In cases where nerve-respecting surgery is performed, there is no significant deterioration in lower urinary tract function, neither clinically nor urodynamically. Even those patients with pathological UD did not experience a deterioration in postoperative status; in fact, the opposite was true. Taking the infiltrative nature of endometriosis into consideration, whilst nerve-respecting surgery may be attempted, it is possible that the infiltration has already caused damage to the IHP unilaterally or bilaterally. The findings of this study are of interest for at least three reasons: Firstly, the patient-reported outcomes in this cohort demonstrated that the LUTS remained unchanged following surgical therapy. In the majority of cases, LUTS were mild to moderate, and, most importantly, they were not clinically significant. Moreover, all symptoms characteristic of endometriosis, as rated on the VAS, exhibited a statistically significant improvement. Secondly, pathological urodynamic findings were present in 20% of the preoperative cohort. The location of the DIE had no significant impact on the results of the filling cystometry or the pressure flow study. Thirdly, postoperatively, there was a trend towards lower uroflow, which, however, lacked clinical significance. In particular, concomitant hysterectomy in conjunction with endometriosis surgery resulted in a statistically significant reduction in uroflow and BCI. In cases where bowel resections were performed, an increase in PVR was observed. However, the increase was not deemed to be clinically significant.

The strength of this study is its simultaneous observation of preoperative and postoperative measurements, in addition to the objective and subjective parameters concerning endometriosis. Patient-reported outcomes (PROs) are of critical importance in the context of urogynecological studies. Accordingly, we employed these validated and reproducible PROs as pivotal elements of this paper. Subjective perception of symptoms frequently does not correlate with the severity of endometriosis. Another strength is the prospective recruitment of patients to the specialized endometriosis consultation and the inclusion of all patients with DIE. Furthermore, the utilization of validated questionnaires and standardized, state-of-the-art urodynamic measurements represent a significant strength.

The principal limitation of this study is the relatively small number of participants, which may have affected the generalisability of the findings. Although the study was designed with a power calculation, the parameters were based on a study of prolapse patients rather than endometriosis patients, which may have introduced confounding factors into the results [30]. The relationship between postoperative LUTD in prolapse surgery and nerve injury remains inconclusive. Nevertheless, our findings indicate that prolapse operations may exert a denervation effect that is comparable to that observed in endometriosis, which we deemed to be an appropriate comparison. It was not possible to include data on endometriosis patients in the power calculation. One potential explanation for this discrepancy is that the postoperative LUTD rate in the existing literature is up to 40% [17], whereas in our collective, it was only 20%. Uroflow was selected as the primary endpoint and measure of bladder function. However, it should be noted that uroflow may be influenced by the current micturition situation, including the use of abdominal pressure, posture, or voiding position. This represents a limitation of the current study. However, secondary endpoints, including BCI and PVR, were also examined and did not indicate any clinically significant bladder function impairment. The application of specific uroflow thresholds (>15 mL/s) to ascertain the probability of impaired bladder emptying is constrained. Furthermore, the presence of reduced uroflow without the concomitant presence of symptoms (such as a slowed urine stream or residual urine sensation) is not indicative of any clinically significant LUTD. A more accessible, continuously available, and cost-effective examination is the sonographic measurement of the PVR. Given the widespread availability, affordability, and non-invasive nature of ultrasound in gynecology, it is recommended to be employed as a routine examination both pre- and postoperatively among patients with DIE. A further limitation is the high number of patients who have undergone previous endometriosis surgery. The majority of previous surgical procedures were conducted in non-specialized hospitals for the treatment of endometriosis.

In contrast with the findings of the present study, a previous retrospective study from our clinic demonstrated a discernible correlation between the DIE nodes in the #ENZIAN compartment B and postoperative voiding dysfunction [17]. Furthermore, previous studies have shown a correlation between bilateral USL resection or rectovaginal dissection and postoperative bladder function [18,35]. Conversely, two prospective studies have demonstrated an improvement in postsurgical bladder function in patients with DIE, particularly during the filling and voiding urodynamic phases [4,36]. Surgical treatment of DIE with meticulous dissection and consideration of neurological anatomy yielded favorable outcomes with respect to lower urinary tract function after surgery. Furthermore, the results of our cohort study indicate that DIE surgery does not appear to cause postoperative LUTS except in cases when combined with hysterectomy or bowel resection. Previous studies investigating the urodynamic findings in patients with DIE have identified higher rates of altered parameters, with some even suggesting that a preoperative urodynamic evaluation should be conducted for all patients undergoing surgery for suspected DIE [4,5,6].

Given the invasive nature of the examination and the considerable expenditure of time, personnel, and costs involved, UD should be performed only in clinically significant situations among patients with DIE and not routinely. It is therefore recommended that preoperative and postoperative sonographic measurements of PVR be employed in this population, as it is a straightforward, widely accessible, and non-invasive measurement. A long-term follow-up would be of great interest, with the aim of assessing the evolution of LUTS and LUTD in our patient population with validated questionnaires and UD, if necessary.

## 5. Conclusions

Surgery for DIE does not deteriorate bladder function. The results of this study indicate that there is no statistically significant difference in uroflow between patients with DIE and those who have undergone surgery. Impaired bladder function is observed among 20% of patients with DIE, both prior to and following surgery. However, UD does not yield isolated, clinically relevant findings, and thus, a measurement of post-void residual is sufficient to detect lower urinary tract dysfunction. Urodynamic testing should not be a routine requirement for patients undergoing surgery for DIE, but sonographic measurement of post-void residual is recommended as an easy, widely available, and non-invasive method.

## Figures and Tables

**Table 1:** Patients’ demographics and surgical characteristics.

Characteristics	Patients (*n* = 51)
Age, yrs, median (range)	34.0 (25–51)
BMI, kg/m^2^, median (range)	23.3 (17–35)
Nulligravida, *n* (%)	36 (70.59)
Nullipara, *n* (%)	47 (92.16)
Wish to have children, *n* (%)	32 (62.75)
Under hormonal treatment, *n* (%)	30 (58.82)
MRI, *n* (%)	29 (56.86)
Previous endometriosis surgery, *n* (%)	19 (37.25)
#Enzian score, *n* (%)	
Compartment A	36 (70.59)
Compartment B	44 (86.27)
Compartment C	25 (49.02)
Compartment FI	6 (11.76)
Compartment FA	13 (25.49)
Compartment FU	2 (3.92)
rASRM stage, *n* (%)	
Stage 1	2 (4)
Stage 2	9 (18)
Stage 3	12 (24)
Stage 4	27 (54)
Duration of surgery, min, median (range)	234 (62–540)
Blood loss, ml, median (range)	174 (10–800)
Surgical procedure, *n* (%)	
Ureterolysis	23 (45.10)
Resection rectovaginal septum	41 (80.39)
Vaginal wall resection	26 (50.98)
Resection uterosacral ligaments	42 (82.35)
Pelvic wall resection	25 (49.02)
Bowel resection	18 (35.29)
Appendectomy	8 (15.69)
Bilateral salpingo-oophorectomy (BSOO)	4 (7.84)
Hysterectomy	10 (20)
Perioperative complication, *n* (%)	2 (4)

Abbreviations: *n* = number, BMI = body mass index, MRI = magnetic resonance imaging. Missing: 0.

**Table 2:** Outcome parameters.

	Preop	Postop	*p*-Value	(95% Cl)	Missing
Urodynamics					
Pressure flow					
Uroflow [mL/s]	22.14	21.53	0.564	(−1.5–2.71)	0
BCI	130.38	116.63	0.046	(0.23–27.27)	10
PVR [mL]	26.1	33.59	0.390	(−24.85–9.87)	0
Filling cystometry					
BV [mL]	356.86	310.22	0.159	(−18.89–112.16)	2
DO [yes/no]	15.69%	4%	0.049	(0.003–0.23)	1
MUCP [cmH_2_O]	95.41	82.08	0.003	(4.76–21.89)	14
Visual analog scale [0–10]					
Dysmenorrhea	5.4	1.75	<0.001	(2.47–4.83)	11
CPP	2.93	1.35	<0.001	(0.79–2.36)	11
Dyspareunia	3.18	0.2	<0.001	(2.09–3.86)	11
Dyschezia	3.18	1.05	<0.001	(1.04–3.21)	11
Dysuria	1.08	0.53	0.109	(−0.13–1.23)	11
IPSS [0–35]	8.37	8.51	0.893	(−2.35–2.05)	10
QoL	1.32	1.29	0.922	(−0.48–0.53)	10
Inc. emptying	1.59	1.37	0.395	(−0.3–0.74)	10
Frequency	1.61	1.22	0.077	(−0.04–0.82)	10
Intermittency	1	1.49	0.079	(−1.04–0.06)	10
Urgency	0.95	0.59	0.117	(−0.1–0.83)	10
Weak stream	0.9	1.44	0.043	(−1.06–−0.02)	10
Straining	0.76	0.90	0.467	(−0.55–0.26)	10
Nocturia	1.56	1.51	0.844	(−0.45–0.55)	10

Abbreviations: Preop = preoperative value; Postop = postoperative value; 95% CI = 95% confidence interval; BCI = bladder contractility index; PVR = post-void residual; BV = bladder capacity; CPP = chronic pelvic pain; IPSS = International Prostate Symptom Score; QoL = quality of life; Inc. emptying = incomplete emptying.

## Data Availability

The raw data supporting the conclusion of this article will be made available from the corresponding author upon request because our data are confidential.

## Supplementary Materials

The following supporting information can be downloaded at: https://www.mdpi.com/article/10.3390/jcm13237367/s1, Table S1: Outcomes of subgroups; Table S2: Preoperative outcomes of subgroups; Table S3: Postoperative outcomes of subgroups.
