# Supplementary material for: Lower Urinary Tract Dysfunction Among Patients Undergoing Surgery for Deep Infiltrating Endometriosis: A Prospective Cohort Study
Source: J Clin Med. 2024 Dec 3;13(23):7367. doi: 10.3390/jcm13237367 (PMC11642162; doi:10.3390/jcm13237367)
Supplement: Supplementary file 1 [file jcm-13-07367-s001.zip › jcm-3329143-supplementary revised.pdf]

Table S1: Outcomes Subgroups

Parameters	Uroflow (ml/s)				BCI				PRV (ml)			
	preop	postop	p-value	(95%CI)	preop	postop	p-value	(95%CI)	preop	postop	p-value	(95%CI)
#ENZIAN score												
Compartment A	21.97	20.97	0.56	(-5.98 - 3.32)	125.09	109.63	0.93	(-31.31 - 28.91)	23.69	37.08	0.29	(-18.02 - 58.13)
Compartment B	21.75	20.84	0.47	(-8.342 - 3.95)	126.59	109.37	0.33	(-61.2 - 21.55)	27.54	32.56	0.48	(32.75 - -17.29)
Compartment C	22.6	20.8	0.26	(-6.54 - 1.8)	134.08	111.6	0.33	(-40.24 - 14.05)	23.52	42.68	0.18	(57.37 - -13.64)
Compartment FI	26.33	27.66	0.5	(-4.37 - 8.77)	161.62	133.4	0.43	(-58.01 - 25.05)	15.5	42.5	0.42	(-31.98 76.2)
Compartment FA	19.46	20	0.53	(-3.32 - 6.4)	114.57	103.3	0.27	(-14.12 - 50.46)	24.46	15.53	0.27	(-61.79 17.73)
Compartments FU	10.5	8.5	0.79	(-12.4 - 9.5)	55	.	.	.	12.5	106.5	**0.04**	(3.42 176.66)
Previous surgery	22.26	22.16	0.72	(-3.59 - 5.19)	129.07	110.5	0.84	(-32.89 - 26.47)	43.63	47.74	0.77	(-41.65 30.86)
Surgical procedure												
Ureterolysis	20.35	22.17	**0.03**	(0.35 - 8.51)	123.37	120.52	0.39	(-15.78 - 39.32)	32.39	36.08	0.69	(28.3 - -12.72)
Resection RVS	22.98	21.21	**0.02**	(-10.94 - -0.77)	130.47	108.62	**0.01**	(-75.48 - -7.84)	22.68	38.39	0.05	(84.43 -10.49)
Vaginal wall resection	21.08	22.73	**0.02**	(0.57 - 8.66)	124.12	112.2	0.57	(-19.73 - 35)	31.12	40.8	0.79	(39.57 - -10.08)
Resection SUL	21.57	20.9	0.9	(-5.91 - 5.24)	126.75	113.3	0.5	(-55.47 - 27.79)	27.4	32.57	0.56	(32.71 - -18.42)
Pelvic wall resection	21	21.96	0.14	(-1.09 - 7.24)	131.8	123.47	0.78	(-23.74 - 31.03)	27.52	41.56	0.46	(47.75 -21.78)
Bowel resection	21.5	21	0.94	(-4.28 - 4.62)	129.18	102.92	0.33	(-44.05 - 15.46)	28.5	54.38	0.11	(64.23 -16.42)
Appendectomy	21.88	26	0.05	(-0.01 - 11.23)	136.83	102.42	0.16	(-60.41 - 10.6)	14.75	34	0.56	(62.03 - -10.56)
BSOO	22.25	19.75	0.6	(-9.94 - 5.83)	144.48	117.75	0.53	(-60.32 - 31.56)	37.5	32.5	0.67	(51.6 - -36.55)
Hysterectomy	17.2	16.4	0.99	(-5.38 - 5.33)	111.84	75.14	0.12	(-62.97 - 7.61)	31.7	18.3	0.22	(17.15 - -9.79)

Parameters	BV (ml)				Pdet (cmH2O)				MUCP (cmH2O)			
	preop	postop	p-value	(95%CI)	preop	postop	p-value	(95%CI)	preop	postop	p-value	(95%CI)
#ENZIAN score												
Compartment A	359.41	331.17	0.35	(-78.02 - 212.64)	24.66	26.33	0.56	(-8.05 - 14.62)	91.06	78.38	0.93	(-19.74 - 18.30)
Compartment B	354.02	292.95	0.45	(-258.46 - 117.98)	25.27	25.64	0.84	(-17.29 - 14.14)	92.59	83.21	0.72	(-22.99 - 32.91)
Compartment C	352.08	298.16	0.86	(-143.76 - 121.25)	26.65	26	0.54	(-13.64 - 7.35)	91.4	72.66	0.07	(-31.47 - 1.8)
Compartment FI	344.33	349.83	0.56	(-141.96 260.78)	23	19.25	0.6	(-21.75 12.78)	106.6	92.5	0.97	(-28.48 27.52)
Compartment FA	254.84	266.76	0.29	(-68.54 227.94)	22.66	28.33	0.23	(-4.9 19.57)	94.22	87.11	0.41	(-11.86 28.28)
Compartments FU	309.5	.	.	.	5	.	.	.	42	.	.	.
Previous surgery	352.26	332	0.41	(-80.82 195.53)	27.28	26.58	0.44	(-15.67 6.93)	92.78	80.42	0.75	(-21.52 15.57)
Surgical procedure												
Ureterolysis	335.7	342.13	0.12	(-29.17 - 230.60)	23.78	23.87	0.698	(-12.72 - 8.61)	87.61	75.66	0.988	(-17.84 - 17.58)
Resection RVS	362.9	315	0.96	(-174.47 - 167.75)	25.91	27.15	0.641	(-10.49 - 16.82)	92.14	78.77	0.634	(-29.07 - 17.95)
Vaginal wall resection	361.73	352.91	0.18	(-42.03 - 217.97)	25.15	26.27	0.929	(-10.08 - 11.01)	85.5	79.17	0.22	(-6.57 - 27.57)
Resection SUL	348.14	295.35	0.93	(-177.86 - 164.34)	25.38	25.61	0.727	(-18.42 - 12.97)	91.16	81.42	0.813	(-24.7 - 31.26)
Pelvic wall resection	356.32	348.39	0.18	(-42.52 - 217.96)	26.82	21.89	**0.016**	(-21.78 - -2.34)	100.09	89.5	0.909	(-18.38 - 16.4)
Bowel resection	312.61	283.23	0.63	(-105.55 - 172.16)	25.77	23.81	0.401	(-16.42 - 6.72)	92.08	79	0.598	(-23.92 - 13.97)
Appendectomy	338	380.75	0.22	(-69.68 - 283.32)	21.29	23.85	0.648	(-10.56 - 16.76)	88	72.57	0.814	(-24.78 - 19.59)

BSOO	281.75	320.75	0.43	(-147.19 - 333.68)	35.75	17.25	**0.012**	(-36.55 - -4.74)	88.25	72.25	0.829	(-30.98 - 24.98)
Hysterectomy	300.9	238.6	0.82	(-185.11 - 147.99)	24.43	28.83	0.515	(-9.79 - 19.1)	85.57	76.66	0.403	(-13.62 - 33.09)

Abbreviations: Preop = Preoperative value, Postop = Postoperative value, 95%CI = 95% Confidence Intervall, BCI = Bladder contractility index, PVR = Postvoid residual volume, BV = Bladder capacity, Pdet = Max. detrusor pressure, MUCP = Max. urethral closure pressure, RVS = Rectovaginal septum, SUL = Sacrouterine ligament, BSOO = Bilateral salpingo-oophorectomy.

Table S2: Preoperative Outcomes Subgroups

Parameters	Uroflow (ml/s)	p-value	(95%CI)	BCI	p-value	(95%CI)	PVR (ml)	p-value	(95%CI)
#Enzian score									
Compartment A	21.97	0.828	(19.52 – 24.42)	125.1	0.436	(107.68 – 142.51)	23.69	0.548	(9.35 – 38.04)
Compartment B	21.75	0.407	(19.19 – 24.31)	126.59	0.444	(111.56 – 141.63)	27.54	0.5587	(13.44 – 41.65)
Compartment C	22.6	0.699	(19.01 – 26.19)	134.09	0.46	(114.24 – 153.93)	23.52	0.684	(5.84 – 41-2)
Compartment FI	26.34	0.189	(17.95 – 34.72)	161.62	0.082	(116.93 – 206.31)	15.5	0.533	(-3.47 - 34.47)
Compartment FA	19.46	0.179	(14.84 – 24.08)	114.58	0.300	(90.29 – 139.86)	24.46	0.878	(-6.11 - 55.03)
Compartments FU	10.5	**0.041**	(4.15 – 16.85)	55	.	.	12.5	0.6582	(-146.33 - 171.33)
rASRM stage									
Stage 1	28.5	**<0.001**	(16.9 - 40.1)	164	**<0.001**	(100.07 - 227.93)	5	0.875	(-58.55 - 68.55)
Stage 2	26.8	0.788	(17.58 - 35.98)	154.99	0.802	(107.45 - 202.52)	41.1	0.306	(3.28 - 78.95)
Stage 3	20.42	0.201	(16.37 - 24.47)	115.35	0.164	(86.31 - 144.4)	19.5	0.673	(-2.34 - 41.34)
Stage 4	20.81	0.205	(17.86 - 23.77)	122.64	0.218	(102.56 142.7)	21.4	0.517	(7.48 - 45.26)
Previous endometriosis surgery	22.26	0.934	(17.55 – 26.98)	129.56	0.96	(102.47 – 155.67)	43.63	**0.026**	(13.89 – 73.37)

Parameters	BV (ml)	p-value	(95%CI)	Pdet (cmH2O)	p-value	(95%CI)	MUCP (cmH2O)	p-value	(95%CI)
Enzian score									
Compartment A	359.42	0.952	(298.08 – 420-75)	24.67	0.32	(20.57 – 28.77)	91.07	0.674	(81.03 – 101.1)
Compartment B	354.02	0.518	(304.59)	25.27	0.416	(21.28 – 29.26)	92.59	0.894	(82.16 – 103.03)
Compartment C	352.08	0.742	(281.4 – 422.76)	26.65	0.658	(21.83 – 31.47)	91.4	0.851	(75.63 – 107.17)
Compartment FI	344.34	0.812	(78.8 – 609.87)	23	0.568	(18.88 – 27.12)	106.6	0.277	(71.1 – 142.1)
Compartment FA	254.85	**0.01**	(168.41 – 341.28)	22.67	0.362	(14.45 – 30.88)	94.22	0.841	(82.2 – 106.24)
Compartments FU	309.5	0.6767	(-700.64 - 1319.64)	5	.	.	42	.	.
rASRM stage									
Stage 1	485	**<0.001**	(236.38 - 733.62)	21	**0.019**	(0.72 - 38.28)	82.5	**0.001**	(36.85 - 128.15)
Stage 2	406.9	0.57	(287.9 - 525.88)	26	0.608	(15.23 - 36.77)	102.9	0.431	(79.71 - 126)
Stage 3	322.6	0.23	(201.7 - 443.64)	26.6	0.547	(15.84 - 37.43)	94..5	0.628	(68.75 - 120.15)
Stage 4	346.1	0.283	(276.71 - 415.43)	25.8	0.593	(21.28 - 30.34)	89.4	0.771	(75.4 - 103.45)
Previous endometriosis surgery	352.26	0.8	(251.75 – 452.77)	27.29	0.563	(20.9 – 33.67)	92.79	0.95	(74.07 – 111.5)

Abbreviations: 95%CI = 95% Confidence Intervall, BCI = Bladder contractility index, PVR = Postvoid residual volume, BV = Bladder capacity, Pdet = Max. detrusor pressure, MUCP = Max. urethral closure pressure, RVS = Rectovaginal septum, SUL = Sacrouterine ligament, BSOO = Bilateral salpingo-oophorectomy.

Table S3: Postoperative Outcomes Subgroups

Surgical procedure	Uroflow (ml/s)	p-value	(95%CI)	BCI	p-value	(95%CI)	PVR (ml)	p-value	(95%CI)
Ureterolysis	22.17	0.576	(18.73 - 25.62)	120.53	0.515	(91.62 - 149.44)	36.09	0.736	(14.57 - 57.6)
Resection RVS	21.22	0.548	(18.92 - 23.52)	108.63	0.163	(90.04 - 127.22)	38.39	0.144	(22.3 - 54.48)
Vaginal wall resection	22.73	0.238	(19.37 - 26.09)	112.2	0.852	(83.11 - 141.29)	40.81	0.27	(19.62 - 62)
Resection SUL	20.9	0.193	(18.62 - 23.19)	113.3	0.873	(95.67 - 130.94)	32.57	0.744	(16.87 - 48.28)
Pelvic wall resection	21.96	0.686	(18.55 - 25.37)	123.48	0.253	(98.32 - 148.63)	41.56	0.242	(17.16 - 65.96)
Bowel resection	21	0.708	(16.91 - 25.09)	102.92	0.386	(64.53 - 141.32)	54.39	**0.019**	(24.06 – 84.71)
Appendectomy	26	0.06	(20.31 - 31.69)	102.43	0.546	(36.21 - 168.65)	34	0.979	(-6.35 - 74.35)
BSOO	19.75	0.619	(5.32 - 34.18)	117.75	0.882	(61.22 - 174.28)	32.5	0.962	(-11.32 - 76.32)
Hysterectomy	16.4	**0.014**	(12 – 20.8)	75.14	**0.036**	(34.9 – 115.38)	18.3	0.253	(6.12 - 30.48)

Surgical procedure	BV (ml)	p-value	(95%CI)	Pdet (cmH2O)	p-value	(95%CI)	MUCP (cmH2O)	p-value	(95%CI)
Ureterolysis	342.14	0.283	(256.02 - 428.25)	23.88	0.278	(16.72 - 31.03)	75.67	0.329	(59.8 - 91.53)
Resection RVS	315	0.709	(256.29 - 373.71)	27.16	0.374	(23.21 - 31.1)	78.77	0.163	(67.54 - 90.01)
Vaginal wall resection	352.92	0.117	(262.24 - 443.59)	26.28	0.969	(20.89 - 31.66)	79.18	0.623	(57.6 - 100.75)
Resection SUL	295.35	0.242	(241.99 - 348.71)	25.62	0.312	(21.6 - 29.63)	81.42	0.73	(69.35 - 93.92)
Pelvic wall resection	348.39	0.18	(260.31 - 436.47)	21.89	**0.019**	(16.68 – 27.11)	89.5	0.18	(74.16 - 104.84)
Bowel resection	283.24	0.465	(225.18 - 341.29)	23.82	0.406	(16.7 - 30.93)	79	0.713	(56.06 - 101.94)
Appendectomy	380.75	0.245	(253.9 - 507.6)	23.86	0.543	(14.4 - 33.31)	72.57	0.398	(86 - 100.28)
BSOO	320.75	0.908	(76.7 - 564.8)	17.25	0.104	(8.59 - 25.9)	72.25	0.53	(21.61 - 122.89)
Hysterectomy	238.6	0.189	(115.74 361.46)	28.83	0.583	(14.56 - 43.11)	76.67	0.662	(30.02 - 123.31)

Abbreviations: 95%CI = 95% Confidence Intervall, BCI = Bladder contractility index, PVR = Postvoid residual volume, BV = Bladder capacity, Pdet = Max. detrusor pressure, MUCP = Max. urethral closure pressure, RVS = Rectovaginal septum, SUL = Sacrouterine ligament, BSOO = Bilateral salpingo-oophorectomy.
